# Youth-Centered Mobile Intervention (Next4You) to Promote Healthy Relationships and Sexual Wellness Among Adolescents in or Transitioning From Foster Care: Protocol for a Randomized Controlled Trial

**DOI:** 10.2196/77185

**Published:** 2026-02-03

**Authors:** Pamela M Anderson, Seow Ling Ong, Dallas Elgin, Jennifer Laird, Darriya Starr, Sade Daniels, Jakara Rogers, Karin K Coyle

**Affiliations:** 1 Education Training and Research, 500 Westridge Drive, Suite 102, Watsonville, CA, 95076, United States, 1 8314402132; 2 RTI International, Research Triangle Park, NC, United States; 3 Dae Dreams Affirmed, LLC, Douglasville, GA, United States

**Keywords:** adolescents, sexual health, technology, randomized controlled trial, mobile app

## Abstract

**Background:**

National birth rates among adolescents have consistently decreased since 1991, yet substantial disparities remain, particularly among youth in foster care, who experience higher risks of unintended pregnancies and sexually transmitted infections. Few sexual health programs rarely address the specific needs of foster youth or incorporate youth perspectives into their design, development, and implementation.

**Objective:**

This study aims to refine, pilot-test, and evaluate Next4You, a fully mobile, youth-centered sexual health education platform tailored specifically for adolescents in or transitioning from foster care. The program intends to reduce sexual risk behaviors and related psychosocial outcomes by promoting healthy relationships, sexual wellness, contraception use, communication skills, self-respect, and wellness education.

**Methods:**

Next4You is a fully mobile, self-paced, 4-week intervention evaluated using an individual-level randomized controlled trial involving a target sample of 500 youths aged 16‐19 years with current or previous foster care experience in California. Participants are randomly assigned to either the intervention group, which receives access to Next4You modules, or the comparison group, which accesses general health materials through a similar web platform. Both groups complete baseline, 3-month, and 9-month follow-up surveys. Immediately following the 9-month follow-up survey, participants in each group receive access to the platform they were not initially assigned. Data collection assesses contraceptive behaviors, sexual communication, consent self-efficacy, and knowledge related to health rights and financial literacy. Intervention engagement is tracked through platform analytics, and qualitative interviews supplement data collection. Data analysis will adhere to intent-to-treat principles, using multilevel regression models to assess impacts.

**Results:**

Funding was awarded in 2021, with institutional review board approval in October 2022. Intervention development lasted approximately 7 months, engaging foster youth in a co-design process to ensure relevance and cultural competence. Study recruitment began in September 2023, continuing until May 2025. Final data collection is anticipated by March 2026, followed by data analysis.

**Conclusions:**

Next4You presents an innovative approach to sexual health education by addressing critical gaps in sexual health education for foster youth through mobile technology. If effective, Next4You could provide another evidence-based option for promoting sexual health among foster youth, guiding policymakers and practitioners in adopting similar trauma-informed and youth-centered interventions.

## Introduction

### Background and Rationale

National birth rates for adolescent women have continuously declined since 1991, falling to 5.6 per 1000 for 15‐ to 17-year-olds (approximately an 85% decrease) and to 25.8 per 1000 for 18‐ to 19-year-olds (approximately a 73% decrease) by 2022 [1]. This improvement has been attributed to a variety of factors, including increased use of contraception facilitated by improved access to clinical services, publicly funded adolescent pregnancy prevention programs, and comprehensive sexual health education [2,3]. Despite this progress, substantial disparities in birth rates persist by race, ethnicity, socioeconomic status, geographic location, and some populations [1]. In particular, foster youth are at a higher risk for unplanned pregnancy and sexual risk behaviors that lead to sexually transmitted infections (STIs). One in three young women in foster care becomes pregnant at least once by age 17‐18 years, with more than half experiencing a pregnancy by age 19 years, twice the rate of their peers not in foster care [4-6]. Young men in foster care are also at high risk for getting someone pregnant by age 19 years [4]. Youth in foster care are more likely than their peers to have had sexual intercourse, to have engaged in transactional sex, and to not have used birth control at last sex [6]. While 70% of pregnant young women in foster care did not plan their pregnancy, only a quarter of them were using birth control when they became pregnant [6], demonstrating a clear gap in prevention services reaching vulnerable youth [7-9]. Youth in foster care may be using birth control inconsistently or incorrectly because they experience instability in access to sexual health information and services, such as changes in caseworkers and living arrangements, and a lack of coordination among agencies providing care [4,5,10]. Additionally, many vulnerable youth have concerns about provider attitudes, privacy, and stigma when they access services [8]. LGBTQ youth, who are disproportionately represented in foster care and have an increased risk for pregnancy and STIs, face additional challenges in accessing health services and education that are inclusive and represent LGBTQ youth’s experiences, relationships, and identities [8,11,12].

For youth in foster care, there are few sex education programs designed specifically for them, and none that are evidence-based and available through the teen pregnancy prevention evidence review use a fully technology-based approach [13]. *Power Through Choices* was the first sex education program designed exclusively for foster youth to be evaluated by a randomized controlled trial [14]. This 10-session program used interactive methods to support youth in developing critical knowledge and sexual decision-making skills. Youth participating in the program were less likely to engage in sexual intercourse without birth control in the past 3 months at 6-month follow-up and had significantly lower odds of ever being or getting someone pregnant at 12-month follow-up than control youth. This program demonstrates that sexual health education can be implemented effectively with foster youth in nonclassroom settings. However, similar to other traditional sexual health education programs, there are limitations to a lengthy, multisession program among a population where high instability is associated with increased risk of pregnancy [5]. And when vulnerable youth cannot access information from trusted sources such as educators and providers, they may turn to the internet [12]. In a nationally representative survey, more than 80% of adolescents reported that they have sought health advice on the internet, with 34% modifying their behavior based on what they found [15]. Mobile technology is almost universally accessible—95% of adolescents have access to a smartphone regardless of gender, race and ethnicity, and socioeconomic status, and the majority (89%) are accessing the internet at least several times a day [16]. Yet, to our knowledge, there has been only one fully mobile sexual health intervention for young people that has been rigorously evaluated, and none that have been designed and evaluated specifically for youth in foster care [13,17].

Engaging youth in the design of interventions that impact their health and well-being is believed to support young people’s development [18], strengthen programs and services [19], and lead to social, political, and economic benefits [20]. Engagement in these processes is also what young people want [19]. Yet, sexual health interventions are rarely designed in collaboration with young people, especially those from systemically impacted communities such as youth in foster care [21]. Additionally, many existing sexual health interventions that meaningfully involve youth lack rigorous evidence on their ability to engage youth and impact health outcomes [21].

Young people, including those in foster care, routinely identify relationships and relationship health as important areas of focus [22]. Romantic relationships play a significant role in adolescent development [23]. By middle adolescence, most young people have been involved in at least one romantic relationship [24], a context in which most sexual interactions occur [25]. Adolescence is a period where young people begin to develop standards and expectations for relationships and build their communication skills. For youth in foster care, these tasks may be more difficult if they have experienced contexts where neglect, aggression, and violence are modeled [26]. Thus, youth in foster care are at increased risk for experiencing intimate partner violence and, because of prior trauma, may be more vulnerable to negative health impacts from intimate partner violence [27]. Yet, developmental psychologists emphasize that relationships developed during adolescence represent a new period where past models of relationships may be reshaped [23], an incredible potential to change unhealthy models and carry positive ones into adulthood, underscoring the potential impact of this content on adolescents’ well-being.

### Study Objectives and Research Questions

The primary study objectives are as follows: (1) refine and update the Next4You mobile responsive platform using trauma-informed and healing-centered principles, centering the voices of youth in foster care and engaging them directly in development; (2) pilot test the updated intervention and planned evaluation activities with youth representing the priority population and finalize based on pilot data and insights from youth and program partners; (3) assess the effectiveness of the new intervention using a randomized controlled trial; (4) develop an intervention manual and implementation package (eg, logic model, core components, adaptation guidelines, and fidelity tool) to support replications; and (5) collect other implementation data and document lessons learned throughout the study.

Textbox 1 summarizes the primary and secondary research questions for the study.

Textbox 1. **Primary and Secondary Research Questions**

**Primary research question**

*Three months following the intervention:*

1. What is the effect of the 4-week Next4You intervention on the prevalence of choosing abstinence or using condoms among participants who had vaginal or anal sex in the past 3 months?

**Secondary research questions**

*Nine months following the intervention:*

1. What is the effect of the 4-week Next4You intervention on the prevalence of choosing abstinence or using condoms among youth in foster care who had vaginal or anal sex in the past 3 months?

*Three and nine months following the intervention period:*

2. What is the effect of the 4-week Next4You intervention on rates of condomless vaginal or anal sex?

3. What is the effect of the 4-week Next4You intervention on sexual consent self-efficacy?

4. What is the effect of the 4-week Next4You intervention on sexual communication self-efficacy?

5. What is the effect of the 4-week Next4You intervention on condom use attitudes?

6. What is the effect of the 4-week Next4You intervention on contraceptive use attitudes and beliefs?

7. What is the effect of the 4-week Next4You intervention on experiences with equity and power balance in relationships?

8. What is the effect of the 4-week Next4You intervention on knowledge of health care rights and location of services?

9. What is the effect of the 4-week Next4You intervention on contraceptive use (specifically experience with condom errors)?

10. What is the effect of the 4-week Next4You intervention on knowledge of educational rights and programs?

11. What is the effect of the 4-week Next4You intervention on financial literacy knowledge?

## Methods

### Study Design Overview

**Figure 1.**
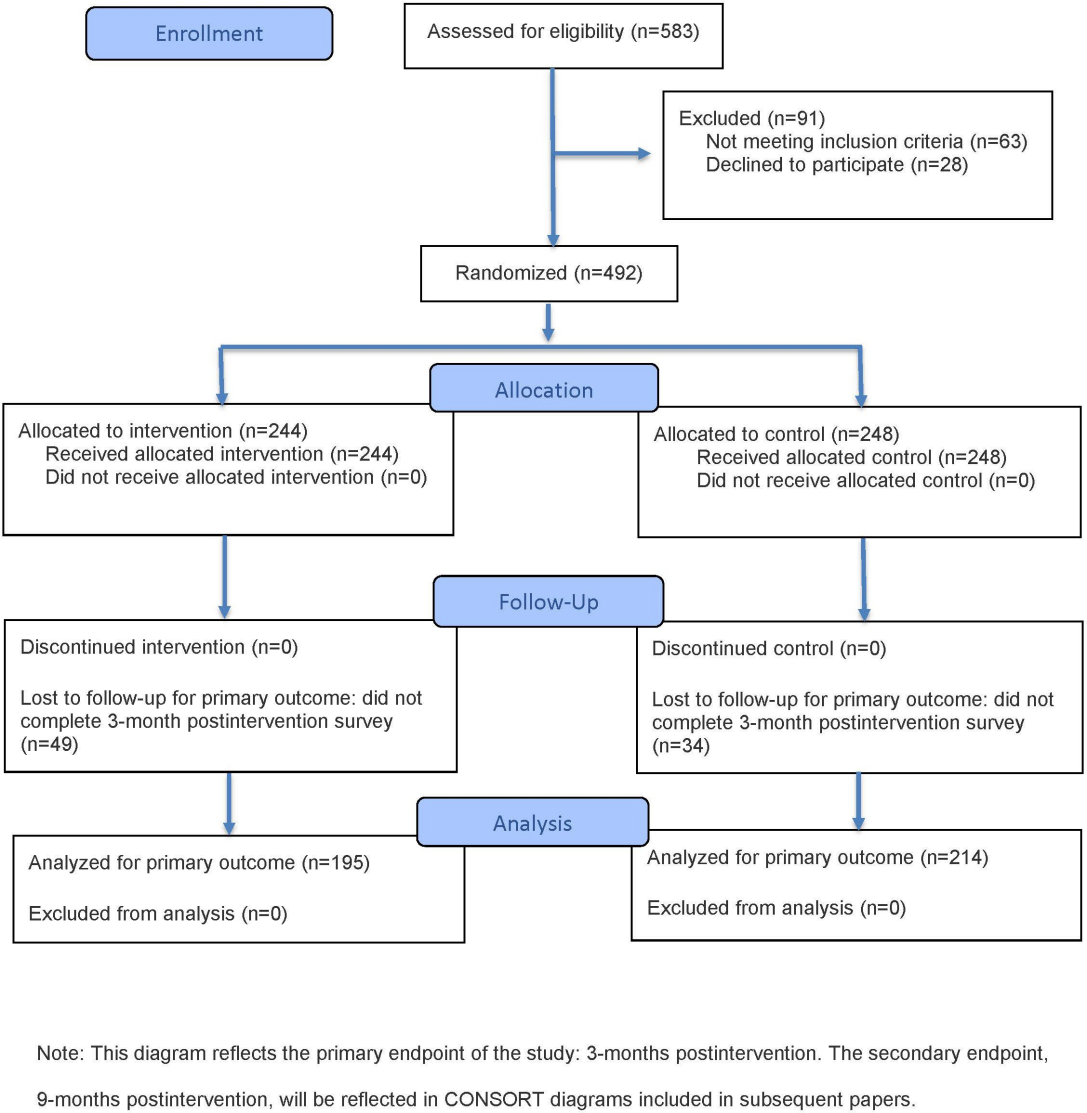
CONSORT (Consolidated Standards of Reporting Trials) diagram for the Next4You study.

Next4You will be tested using an individual-level (randomized controlled trial) involving up to 500 young people ages 16‐19 years with former or current experience in the California foster care system. Half will be assigned to an intervention group and the other half to a comparison group. The study survey will be collected at three time points: baseline (ie, after assent and consent and before randomization) and 3 and 9 months after a 4-week intervention period (applied to both the intervention and comparison groups). The evaluation of Next4You was designed to meet the Institute of Education Sciences’ *What Works Clearinghouse* (WWC) design standards without reservations [28]. Figure 1 presents the CONSORT (Consolidated Standards of Reporting Trials) flow diagram illustrating participant enrollment, allocation, follow-up, and analysis for the study.

#### Study Setting

The study will be completed digitally, among participants who have been referred to the study through partner organizations in 24 counties throughout California.

#### Eligibility Criteria

Youth must meet the following criteria to be eligible for the study: (1) age 16 to 19 years at time of study enrollment; (2) currently in foster care or formerly in foster care (and were in foster care on or after their 13th birthday); (3) currently residing in California; (4) proficient in English; and (5) have access to a tablet, laptop, desktop computer, or phone that is Wi-Fi- or cellular-enabled.

They also had to be free of severe mental health issues and have the cognitive ability to meaningfully engage with the web platform as determined by the recruitment ambassadors.

### Intervention and Comparison Protocols

#### Intervention Condition

Participants randomized to the active intervention condition will receive access to Next4You, a fully mobile intervention designed to reduce rates of unintended pregnancy and STIs among young people currently or recently in the California foster care system. The platform was designed in partnership with foster care youth and with input from community-based organizations serving this population. The responsive, web-based platform can be accessed by young people at any time from an internet-enabled mobile device.

The intervention features 6 content modules (Table 1): Sexual Wellness, Contraception, Relationships, Respecting Self and Partners, Communication, and Building Wealth and Wellness (eg, financial literacy, educational rights, and mental health), with a total instructional time of approximately 3 to 4 hours. Each module includes 5‐12 microlessons, designed as brief, informal snippets of content that are 5 minutes or less; focused on a single learning concept; and are delivered through diverse approaches such as games, videos, quizzes, and others [29]. One advantage of this method includes the repeatability of the content in time, place, and format preferred by most young people [30]. In addition to microlearning, the site includes gamification features, a youth-designed retention strategy to increase motivation to engage youth in the content.

**Table 1.** Intervention modules.

Module	Microlessons, n	Brief description of module content
Sexual Wellness	9	Defining sexual wellness; sexual identity and orientation; sexually transmitted infections and ways to reduce risk; using and accessing health care to maintain sexual wellness; sexual boundaries; sexual and reproductive health rights; sexual anatomy and tips for increasing pleasure.
Relationships	10	Elements of a safe and secure relationship; factors that suggest it may be time to end a relationship; tips on navigating a breakup; navigating loss and feelings of rejection after a relationship ends; intimate partner violence; love bombing; strengthening and building connection in relationships; exploring factors that make young people with lived experience in foster care more susceptible to trafficking; exploring power and control behaviors that may show up in relationships.
Communication	7	“I” versus “you” statements; preparing for having difficult conversations; strategies for managing emotions that may surface during difficult conversations; strategies for de-escalating conversations; strategies for active listening to improve communication skills.
Respecting Self and Partners	7	Self-love and self-worth; affirmative consent; what respecting a partner includes; personal boundaries; strategies for navigating different personal boundaries; gender and gender identity.
Contraception	5	Condom use and steps; common methods of birth control; withdrawal from a harm reduction perspective; contraception rights; options if methods of contraception fail.
Building Wealth and Wellness	12	Passion and purpose; educational rights; college transition and support services; financial rights; coping skills; financial aid options; trauma—building resilience and navigating past trauma; mental health; budgeting; body image.

The Next4You platform includes a total of 50 microlessons, and participants earn points for each completed lesson. To motivate early engagement with the website, participants receive a US $25 e-gift card upon completing 5 lessons. Subsequent points are banked, and the remaining incentive amount is distributed at the end of the 4 weeks, based on the total amount of points earned. Participants may earn up to US $100 in e-gift cards for completing all 50 microlessons. In addition to lesson completion, participants can earn up to US $25 in bonus incentives through all 4 achievement badges: Start Up, On Fire, My Faves, and Consistency. In total, participants may earn a maximum of US $125 in e-gift cards over the 4-week program period.

The online platform will collect paradata in the form of timestamps and counts of user sessions for all actions performed by each user, thereby allowing for calculation of the time spent engaging with the intervention (including total time spent on each microlesson, modules, and engaging with various activities and resources), frequency of logins, and the amount of completed content. Users will be automatically logged out after approximately 10 minutes of inactivity to protect privacy and reduce the likelihood of inflated duration data. The evaluation will use the duration data to conduct exploratory analyses examining differing levels of exposure and the relation to study outcomes.

#### Intervention Conceptual and Development Frameworks

The development of the Next4You intervention draws upon other pertinent knowledge bases to center youth voice through a youth participatory process and draws on the latest research in positive youth development and microlearning to strengthen the intervention’s content and strategies (see the screenshots in Multimedia Appendix 1). Positive youth development is a strength-based approach used to promote adolescents’ prosocial competencies and build skills related to their positive health and well-being [31]. The adolescent development literature guided Next4You’s relationship development content and helped ensure it is age appropriate [23]. The intervention also draws on dual process theories to address the social-emotional and cognitive influences on sexual decision-making [32]. Finally, the intervention draws from social cognitive theory both in terms of key constructs in skill acquisition, such as building self-efficacy or confidence in one’s ability to perform a skill, and in shaping the process of learning, such as through using observational learning or modeling during instruction [33].

#### Intervention Co-Design Process

Engaging youth with lived experience in the foster care system as subject matter experts was an essential first step in developing the Next4You platform and its content. This approach provided a unique opportunity to deliver accurate, inclusive, culturally competent, and on-demand information, resources, and tools tailored to the needs of transition-age youth. To refine and enhance the platform before piloting, the team used youth participatory methods to engage these subject matter experts. These methods incorporate positive youth development principles, emphasizing the importance of engaging young people as partners by respecting their knowledge, insights, expertise, and leadership [34]. Recognizing that youth in foster care often experience trauma, we used trauma-informed and healing-centered principles when engaging co-designers, including safety, equity, empowerment, and resilience. This ensured that the co-design process was inclusive, culturally relevant, and supportive, even when difficult topics arose.

The co-design process involved 9 participants, ages 19‐22 years, from California, who engaged individually and as part of a group over 7 weeks through weekly 90-minute meetings. The facilitation approach was intentionally structured yet flexible. The sequence included 2 sessions on discovery (eg, community building), 2 on defining (research and product design and function), and 3 on designing content for intervention modules, concluding with a design and testing session. Slide decks structured each meeting, but sessions were highly interactive, incorporating icebreakers, small-group discussions, and digital tools (eg, Mentimeter or Kahoot). Participants were invited to be active cocreators rather than passive reviewers; facilitators used semistructured prompts such as “What keeps users coming back?” or “What does wellness mean to you?” to elicit ideas that shaped both content and user experience. Through this process, participants influenced the platform’s esthetics, navigation, and functionality, including customizable color schemes, authentic imagery, gamified features, and microlesson delivery. They identified priority content areas and co-developed stories, scenarios, and audio narration reflecting real experiences. The team used mockups and mood boards to align design decisions with participant input and maintained engagement between sessions through asynchronous channels (eg, GroupMe). Following co-design, pilot testing of 18 lessons yielded highly positive feedback. Participants recommended adding more interactive activities, videos, and visual animations, as well as expanding life-skills content such as improving communication with adults in positions of authority including teachers and supervisors. These refinements strengthened both usability and relevance for youth transitioning from foster care.

By centering youth as co-designers, the Next4You project produced a platform that authentically reflects their experiences and priorities, demonstrating the power of participatory design to advance youth-driven innovation in adolescent health.

#### Comparison Intervention

Youth randomized to the comparison condition will have access to a mobile-responsive website featuring digital resources focused on nutrition, sleep, stress and anxiety, and exercise. Consistent with the intervention group, participants in the comparison group will have unlimited access to the general health digital resources for the initial 4-week period. Throughout the evaluation, youth assigned to the control condition will also continue to receive the standard resources and services typically available to them through their involvement in the foster care system. The comparison condition was selected to establish an effective treatment contrast [35] by isolating the difference between the treatment received by youth with access to the Next4You platform and the treatment received by youth who instead received access to digital resources focusing on nutrition, sleep, stress and anxiety, and exercise. After the initial 4-week intervention phase, comparison group participants will retain access to the comparison website. Upon completion of the 9-month evaluation period, these participants will additionally receive access to the Next4You intervention website for the remainder of the study, concluding on September 29, 2026.

### Sample Size and Power Analyses

Our proposed sample of 500 young people (with a 1:1 allocation to each condition) would result in the Next4You evaluation being powered to identify effects in the range of small to “substantively important” (ie, 0.25 SDs, in accordance with the WWC standards) [36]. The power analysis estimates were obtained and validated via Optimal Design Plus Empirical Evidence software and the HHS Office of Adolescent Health’s Teen Pregnancy Prevention MDI and MDES calculator. The estimates were developed based on the following parameters in accordance with the funder evaluation standards: simple randomization at the individual level; 25% attrition rate; type I error rate of 5%; type II error rate of 20% (ie, power of 0.80); 2-sided hypothesis test; 50% probability of assignment to the treatment group; outcome prevalence of 50%; 25% of the outcome variance is explained by covariates. The impact model will include the baseline measure of the outcome and age or grade level, sex, and race and ethnicity as covariates consistent with funder requirements. The study team will use a combination of data- and theory-driven approaches to select additional covariates for inclusion in the model to further improve the precision of the impact estimates.

### Recruitment

Youth will be recruited from community and school programs serving young people in or transitioning from foster care. Recruitment and enrollment will occur on a rolling basis during years 2 and 3 and the first quarter of year 4 across 24 California counties, including postsecondary on-campus support programs such as Guardian Scholars and NextUp. Each collaborating site designates a recruitment ambassador, typically an adult supporter who works directly with foster youth. The primary criterion for ambassadors is that they are staff members who meet individually and have direct contact with youth who meet study eligibility criteria. The study team trains all recruitment ambassadors and provides them with a standardized protocol that includes approved materials (recruitment flyers, brief videos explaining the study, a frequently asked questions document, and recruitment scripts). Upon completion of the training, ambassadors promote the study at their site, share the opportunity with youth who come in for direct services, and incorporate the study into the site’s normal outreach mechanisms (eg, newsletters, direct mailing, and virtual and hard copy posters with a QR code). Recruitment ambassadors are trained to only present the study opportunity to youth who meet the study criteria based on their knowledge of the young people. Youth enroll in the study through an enrollment portal at partner sites (with ambassadors supporting youth through the process), which involves completing a screener and reviewing and signing the study consent form. Youth are also asked to input a recruitment code (which is unique to each recruitment ambassador), which allows the study team to track recruitment and confirm eligibility.

### Consent and Assent Process

We received a waiver of parental permission from the ETR institutional review board for youth ages 16‐17 years, who will complete an assent form written at an eighth-grade reading level, while youth ages 18‐19 years will complete a consent form at the same readability level. Both forms are collected online to align with the fully mobile nature of the intervention. The assent and consent forms include 3 multiple-choice questions to assess young people’s understanding of what they are being asked to do. If youth mark a question incorrectly, a clarifying message will be provided to ensure they have the information needed to make their decision. At the end of the consent form, youth will be asked to indicate their decision of whether they want to take part or not or if they would like to talk with a study team member to get more information before deciding. Youth who decline participation will be thanked for their consideration and will not be contacted again. Youth who say they would like to talk with a study team member will be contacted by a study team member within 2 business days to provide clarifying information. These youth would then be allowed to make their decision online.

### Randomization

Randomization takes place after young people submit their baseline survey using the randomizing feature in Qualtrics with a 50‐50 allocation fraction. More specifically, 50% of participants will be allocated to the intervention group and 50% of participants will be allocated to the comparison group. The study evaluator oversees and manages the randomization protocol. Once randomized, youth are automatically re-directed to the Next4You log in page, based on their assigned conditions along with steps for logging in and establishing their accounts.

### Data Collection Methods

#### Baseline Survey

The evaluation of Next4You will rely on a confidential youth self-report survey assessing knowledge, attitudes, beliefs, and behaviors related to sexual health through web-based surveys administered via the Qualtrics platform. Young people receive a unique link via email to the survey immediately after affirming their assent to study participation. The study team will follow up with youth who do not complete the baseline survey by sending up to 3 texts and 2 additional emails over the course of approximately 3 weeks, each with the unique link to their baseline survey, and an offer to answer questions or support in completing the survey. The study team will then engage the recruitment ambassador for assistance in reaching out to the youth and encouraging them to complete the survey.

Participants will be randomized after the baseline survey and are automatically re-directed at the end of the survey to access their assigned intervention condition. The baseline survey is estimated to take approximately 25 minutes to complete. Participants receive a gift card worth US $40 for filling out the baseline survey.

#### Preparing for Follow-Up Surveys

One month prior to sending each follow-up survey, youths are asked to provide the study team with their latest contact information. Each youth is contacted simultaneously at their last known phone number and email address—one of which has usually not changed in the 3- and 6-month periods since their last follow-up survey. This outreach serves to both remind youth that a follow-up survey will be sent to them in the next few weeks and maintain the most recent, preferred contact information to reach the youth when their next survey is sent. Doing so 1 month in advance gives youth time to reply or receive up to 3 reminders if needed. This confirmation of contact information request is associated with a US $10 e-gift card when the youth replies to the study team with their current contact information.

#### Follow-Up Surveys (3 and 9 Months)

Both follow-up surveys are administered online using procedures like those used at baseline. The study team will use a multimethod outreach process to contact youth who do not respond to the surveys. In the first phase, the study team will send up to 3 simultaneous texts and emails to encourage nonrespondents to complete the survey. During the second phase, youth who have not responded will receive up to 2 calls (with voicemails) from the study team during the following week. If youth have not completed the survey during the second phase, the study team will wait 1 week before implementing the third phase of outreach, which involves engaging with the study recruitment ambassadors for assistance in reaching out to the youth and encouraging them to complete the survey. At all outreach attempts by the study team, participants are provided with the study team’s email and phone for any questions or need for assistance in completing the survey.

#### Implementation Evaluation Data Collection

Given the intervention is fully mobile, our implementation evaluation data collection focuses on four areas related to fidelity that have been found to influence dose and treatment effects in mobile interventions [37]: (1) amount (count of lessons completed), (2) frequency (counts of user sessions and patterns of use), (3) duration (amount of time spent in each module and lesson), and (4) depth (total content and engagement as a percentage of all available content). These data will be collected from the web platform directly. We will also conduct selected postintervention interviews to gather qualitative data on user experiences during the study.

#### Retention

The study team will implement a comprehensive participant tracking and retention strategy designed to maximize response rates and minimize attrition across all follow-up periods. This approach includes allocating sufficient resources for incentives, dedicating staff time to personalize follow-up, and conducting multiple outreach attempts to nonrespondents. Consistent with intent-to-treat (ITT) principles, all youth enrolled in the evaluation will be tracked and will receive retention-focused outreach, regardless of level of engagement or participation.

Participants will receive e-gift card incentives for completing evaluation surveys, verifying or updating contact information, and completing intervention activities. In total, youth may earn up to US $315 for completing all activities. Incentive amounts include US $40 for the baseline survey, US $60 each for the 3- and 9-month follow-up surveys, US $10 per response (up to 2 times) for confirming or updating contact information, and up to US $125 for completing intervention activities. Incentive values were informed by a consultant with lived and professional experience in the foster care system to ensure that compensation reflects and honors participants’ time and expertise.

Retention in the intervention group platform will include gamification features such as badges and a point-based reward system that allows for points to be converted into electronic gift cards. Additionally, push factor text and email notifications will be used to promote engagement. For example, if there has been no activity for at least 3 days, an automatic notification will go out to participants inviting them to log onto the platform and continue earning points through the completion of lessons. Youth assigned to the comparison group platform will receive equivalent total incentives for completing a minimum of 12 online pamphlets [38-49] on promoting healthy eating and nutrition, sleep, exercise, and reducing stress and anxiety. The total amount of incentives that youth may receive is the same for both study groups.

### Outcomes

#### Primary Outcome

The primary outcome of this study is the impact of the 4-week Next4You intervention on contraceptive use among youth in foster care. Specifically, this study will examine the prevalence of participants who report either abstaining from sex or using c condoms during vaginal or anal sex in the past 3 months, compared with participants provided access to a mobile-responsive website containing only general wellness resources (sleep, nutrition, stress and anxiety, and exercise). This outcome will be assessed using data from the 3-month postintervention follow-up survey.

#### Secondary Outcomes

The secondary outcomes will examine the impact of the 4-week Next4You intervention, compared with a mobile-responsive website on wellness topics, on a range of sexual and reproductive health and well-being outcomes among youth in foster care. Specifically, analyses will assess effects on condom use behaviors, condomless sex, and condom errors; self-efficacy related to sexual consent and communication; attitudes toward condom and contraceptive use; power balance and experiences within sexual relationships; and knowledge of health care rights, educational rights, service locations, and financial literacy. Outcomes will be assessed at 3- and 9-month postintervention follow-ups.

Multimedia Appendix 2 [50-57] highlights our primary and secondary measures, with sample survey items.

### Statistical Methods

#### Overview of Analytical Strategy

Our analysis plan follows a series of analytic steps, including data cleaning, analysis of baseline equivalence and attrition, handling of missing data, descriptive analysis, impact analyses of confirmatory research questions, and exploratory analyses. Throughout the analysis phase, we will use a significance level of 0.05 and employ an ITT framework, whereby young people who were randomly assigned to a condition are considered to be in that condition regardless of engagement level or intervention dosage. All analyses will be carried out using Stata. The results of the power analyses suggest a minimum detectable impact between 0.11 and 0.13, or 11 to 13 percentage points, for the dichotomous outcomes. These figures correspond to a minimum detectable effect size of 0.23 to 0.25 SD, or a reduction in continuous outcome from 50% to 37.5%, assuming a baseline prevalence of 50%.

#### Baseline Equivalence

Prior to estimating any outcome models, the study team will follow WWC group design standards related to baseline equivalence, attrition, and missing data [58]. The team will first test for differences between treatment conditions on baseline measures of all outcomes and demographic factors. Following WWC standards, effect sizes will be computed using Hedge *g* and any differences above 0.05 noted. Because of random assignment, group differences on baseline or demographic factors are unlikely, but baseline measures for all outcomes will be included as a covariate in all planned impact analyses, and any significant differences in demographic factors (ie, effect sizes between 0.05 and 0.25 SD) will be added as additional covariates.

#### Attrition

The team will then calculate overall and differential attrition rates. Overall youth attrition rates will consist of the number of units without observed outcome data in the analysis against the number of units randomized. Differential attrition will be obtained by contrasting the attrition rates between treatment and control groups. Overall youth attrition and differential attrition between the treatment and control groups will be carefully monitored to determine the levels of potential bias.

#### Handling Missing Data

Missing data for all youth who agree to participate in data collection will be addressed with regression imputation using Stata, with special attention paid to WWC recommendations (eg, separately imputing data from treatment and control conditions). Regression imputation is the preferred approach for this study as it can be used to include participants with missing baseline data and missing outcome data (whereas nonresponse weighting, another imputation method, can only be used for the former). The team will use imputation regression models that (1) are conducted separately by condition or include an indicator variable for condition, (2) include all covariates used for adjustment in the impact model, and (3) include the outcome when imputing missing baseline data.

#### Descriptive and Impact Analyses

The study team will use simple averages and frequency distributions to describe the prevalence of each outcome for youth overall and for the treatment group, by selected demographic characteristics and by usage estimates, to provide an initial understanding of changes in the primary and secondary outcomes before estimating impact estimates using multivariate models. The impact of Next4You will be estimated under the ITT framework by comparing outcomes for individuals assigned to Next4You and those assigned to the control condition. The sample will include all youth who participated in the baseline survey and completed the 3-month follow-up, 9-month follow-up, or both. The study team will model all outcomes with a linear-based 2-level multilevel regression model with youth (level 1) nested within level 2 (sites) to account for the nested structure of the data, adjust for correlated error terms, and avoid underestimating standard errors and increasing the probability of type I errors. The following 2-level random intercept multilevel model will estimate the impacts of Next4You on the primary outcome with separate models estimated for the 3- and 9-month follow-ups:


$$y_{ij}=\;\beta_{o}+\beta_{1}Treat_{1ij}+\beta_{2}Baseline_{2ij}+\beta_{3}X_{3ij}\ldots +\beta_{p}X_{pij}+u_{j}+e_{ij}$$

where $y_{ij}$ refers to the expected probability of exhibiting the corresponding outcome for individual *i* in site *j*; $\beta_{0}$ is the overall mean of the outcome across all groups; $\beta_{1}{Treat}_{1ij}$ is an indicator equal to one for participants assigned to the treatment group and zero for those assigned to the control group; $\beta_{2}{Baseline}_{2ij}$ is the baseline measure of the outcome; $\beta_{3}X_{3ij}+\ldots +\beta_{p}X_{pij}$ represents a vector of individual-level baseline covariates (such as sex, race and ethnicity, and age); $\mu_{j}$ is a site-level random effect; and $\varepsilon_{ij}$ is an individual-level error term. The estimation of the effects of the primary outcome and other dichotomous outcomes via multilevel linear probability models provides a convenient, alternative approach to logit models for interpreting dichotomous outcomes [59]. In particular, linear probability models provide model results on a probability scale that is easier to interpret, whereas logit-based versions of the models express the findings in terms of log odds that are more challenging to interpret.

The use of a 2-level random intercept multilevel model is contingent upon the results of a likelihood-ratio test comparing the fit of the 2-level model against a single-level linear probability model. The null hypothesis for the likelihood-ratio test is that there is no significant difference between the two models. A *P* value of <.05 for the likelihood ratio test would result in the rejection of the null hypothesis, as there was a statistically significant difference between the models with the 2-level model fitting significantly better than the single-level linear probability model. In the event that the null hypothesis could not be rejected, the study team will use a single-level model.

The team will adjust *P* values to account for multiple hypothesis testing within each primary and secondary outcome domain using the Benjamini-Hochberg procedure [60]. Finally, the study team will conduct sensitivity analyses to test the robustness of the findings to alternative assumptions or specification of the multilevel models. Sensitivity analyses may involve comparing findings between multilevel logistic regression and multilevel linear probability models, assessing the sensitivity of approaches for addressing missing data, and assessing the impact of sibling participation on outcomes.

#### Implementation Data Analyses

User analytics captured directly on the platform will be analyzed to monitor participant engagement with both the intervention and comparison group platforms, exploring user profiles and how engagement may relate to behavioral outcomes in our analyses. Specifically, the study team will use these data to conduct exploratory analyses of dosage and treatment effects as moderators to assess how, if at all, the level of exposure to the platform influences the strength of the observed intervention effect. These data will also be within sensitivity analyses where a “treatment on the treated” analysis framework is used to examine the robustness of the impact estimates under the ITT framework. Various definitions of intervention uptake will be examined, including amount, frequency, duration, and depth among participants who ultimately received the condition to which they were assigned (ie, “compliers”). The effect of treatment receipt for the subgroup of compliers, or the local average treatment effect, will be estimated using instrumental variable methods with a two-stage least squares estimator. In the two-stage least squares, the first stage will involve using the random assignment indicator to predict intervention participation, and the second stage will use the predicted values of intervention participation from the first stage intervention to estimate intervention effectiveness.

#### Ethical Considerations

This study was funded in 2021 through the Family and Youth Services Bureau as part of the Personal Responsibility Education Innovative Strategies program as an award to ETR. This project was approved by the ETR institutional review board in October 2022. The study is registered at ClinicalTrials.gov (NCT06127277).

All participants provided informed consent or assent prior to participation. Youth aged 16-17 years provided assent under a waiver of parental permission, and youth aged 18-19 years provided informed consent. Consent and assent procedures were conducted electronically and designed to ensure participants’ understanding of study procedures, risks, and benefits, consistent with the fully mobile nature of the intervention. Participants were compensated for their time through electronic gift cards for survey completion, confirmation of contact information, and engagement with the intervention activities. Incentive amounts were structured to reflect participants’ time and effort; full details of compensation and retention strategies are described in the Retention section. All consent forms and survey data will be stored securely within the Qualtrics platform, which employs firewalls, encryption, and independent security audits to protect participant information. Data will be accessible only to authorized project staff using password-protected, two-factor-authenticated devices. Personally identifiable information, such as names or email addresses, will be stored separately from survey responses, and each participant will be assigned a unique study ID used in all analyses. No identifiable information will appear in reports or publications.

## Results

The study was funded in 2021, with ETR institutional review board approval in October 2022. The Next4You intervention was built over a period of 6‐8 months. Study recruitment launched in September 2023 and completed in May 2025, with final follow-up surveys expected to be completed by March 2026, at which time we will initiate final study analyses. The study ends in September 2026.

## Discussion

### Strengths and Limitations

Adolescents in foster care experience disproportionately higher rates of teen pregnancy and STIs compared to their peers [61]. Despite these disparities, there are few evidence-based sexual health programs tailored for youth in foster care [62], a critical gap given their sexual health needs and the disparities they are navigating [61]. Further, existing evidence-based programs for these young people, like *Power Through Choices* [14] and *Making Proud Choices* (adapted for systems involved youth) [63], are delivered via group-based instruction, which may limit young people’s ability to access content when they need it. A recent meta-analysis focused on digital sexual health promotion interventions found that most of the studies reviewed had a positive impact on cognitive perceptions and sexual behavior outcomes, highlighting the potential for digital interventions [64].

The Next4You study addresses current gaps in sexual health promotion for young people in foster care by testing a fully mobile sexual health program created for foster youth and informed by their lived experiences, allowing young people to access content they identified as key and return to it when they want or need it. Our co-design process actively engaged young people, ensuring the intervention was relevant, authentic, and tailored to their specific needs. A “nothing about us without us” approach not only makes the content more engaging and relatable for participants, but also represents an innovative, trauma-informed methodology that is rarely implemented in adolescent sexual health interventions [65]. The content identified by Next4You co-designers aligns with the findings reported by Aparicio et al [22], with microlessons focused on content from all 3 core theoretical categories identified by foster youth and foster care staff they interviewed (reproductive system health, relationship health, and mental health).

To illustrate, a core focus of the Next4You intervention is its concentration on fostering strong relationships—examining their characteristics, exploring power and control and how that may show up in relationships, and learning and practicing communication skills to enhance communication without abuse, among other topics. This content was deemed as most relevant by the co-designers and is taking a more prominent role in sexual health programs now than in the past, which aligns with adolescent developmental needs [66]. Nonetheless, most existing evidence-based sexual health programs include limited coverage on healthy relationships, such as single lessons or activities on healthy and unhealthy elements of relationships. This is insufficient to help youth build healthy relationships, end unhealthy ones, and learn skills to navigate and communicate personal sexual boundaries and negotiate contraceptive use with a partner. *Love Notes* [67], one of the few healthy relationship education curricula for youth, highlights the potential of a more focused approach; however, there remains a significant gap in the current evidence base, particularly for young people in foster care settings. The Next4You study will address this gap and examine the impact of a relationship-based approach for youth with experience in foster care.

Next4You was designed using a fully mobile, self-paced delivery format because digital health interventions can offer important advantages for adolescents, such as greater accessibility, privacy, and personalization of content [64]. Next4You will allow young people to engage with the content at their own pace, on their own time—a flexibility that is especially valuable given the myriad demands on young people’s time. The gamified, interactive system is expected to sustain youths’ interest and facilitate learning in a youth-friendly way. The mobile format of Next4You is anticipated to lower many traditional barriers to implementation, complementing services provided by existing organizations and educational programs providing direct services and support to foster youth.

Despite these strengths, there are important limitations to consider. A self-paced digital intervention relies on adolescents’ motivation and engagement. Foster youth juggling the stresses of placement changes, trauma, and daily life may not consistently complete online modules without encouragement, despite the incentives that we provide for participation. The absence of a live facilitator means there is no built-in adult support to answer questions in real time or to prompt participation if a young person loses interest. Fully mobile programs also require access to devices and Wi-Fi that could create barriers for some, though most young people have phones [16], and there are programs that provide support for home internet, phones, and data plans (eg, California LifeLine Foster Youth Program and FCC LifeLine Program). It is also possible that some sensitive topics could elicit emotional reactions, given participants’ trauma histories. In anticipation of this, we have included resources for follow-up and are using a recruitment model that is anchored at service organizations so that young people have options for follow-up support. This is an area of focus in exploring participants’ reactions to the program. Further, Next4You was developed expressly for youth involved in the California foster care system; therefore, findings and program components may not be directly generalizable to foster youth in other states. Further, although this study was sufficiently powered to detect a small effect (approx. 0.25 SDs, considered substantively important per WWC criteria) [58], it may have limited power to detect smaller yet potentially meaningful intervention effects. More broadly, while digital interventions show promise in improving adolescents’ sexual health knowledge and attitudes, the evidence base for fully self-paced programs is still emerging, underscoring the value of studies like this one. In summary, the innovative, youth-driven design and scalable digital platform of Next4You are major strengths, but these strategies are not without potential challenges.

### Conclusions

The Next4You intervention represents an innovative, youth-centered approach to addressing sexual health disparities among foster youth. If proven effective, Next4You could provide an accessible and scalable solution to address a critical gap in sexual health education for this group of young people. Policymakers and practitioners could leverage this approach to normalize sexual health conversations, reduce stigma, and ultimately empower foster youth to make informed, healthy decisions.

## Supplementary material

10.2196/77185 Multimedia Appendix 1 Screenshots of the intervention.

10.2196/77185 Multimedia Appendix 2 Study outcome measures.

## Abbreviations

CONSORT: Consolidated Standards of Reporting Trials
ITT: intent-to-treat
STI: sexually transmitted infection
WWC: What Works Clearinghouse
