# Supplementary material for: Youth-Centered Mobile Intervention (Next4You) to Promote Healthy Relationships and Sexual Wellness Among Adolescents in or Transitioning From Foster Care: Protocol for a Randomized Controlled Trial
Source: JMIR Res Protoc. 2026 Feb 3;15:e77185. doi: 10.2196/77185 (PMC12867466; doi:10.2196/77185)
Supplement: Multimedia Appendix 1 [file resprot-v15-e77185-s001.pdf]

Next4You

Welcome to Next4You!

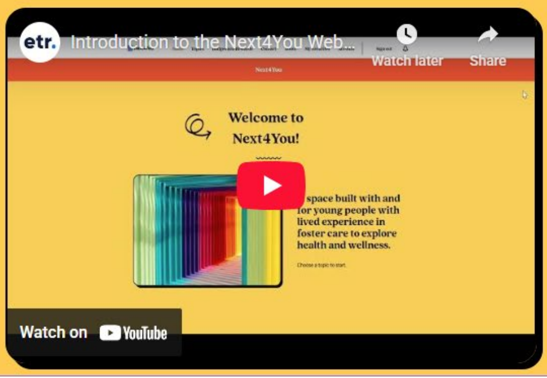

A space built with and for young people with lived experience in foster care to explore health and wellness.

Explore the topics below!

The Topics

View All

Communication

Explore how to use communication strategies to center your power.

Earn 14,000 points

Relationships

Reflect on and explore different aspects of romantic relationships - both safe and less safe.

Earn 20,000 points

Sexual Wellness

What does it mean to you? Explore different aspects of sexual wellness.

Earn 18,000 points

Contraception

Check out options for staying safe from sexually transmitted infections and/or unplanned pregnancy.

Earn 10,000 points

Respecting Self & Partners

Reflect on and explore different aspects of romantic relationships - both safe and less safe.

Earn 14,000 points
