# Supplementary material for: Youth-Centered Mobile Intervention (Next4You) to Promote Healthy Relationships and Sexual Wellness Among Adolescents in or Transitioning From Foster Care: Protocol for a Randomized Controlled Trial
Source: JMIR Res Protoc. 2026 Feb 3;15:e77185. doi: 10.2196/77185 (PMC12867466; doi:10.2196/77185)
Supplement: Multimedia Appendix 2 [file resprot-v15-e77185-s002.docx]

# Study Outcome Measures

| **Impact Research Question** | **Outcome (Domain)** | **Measures** | **Intended Respondents** | **Psychometric Information** | **Citation(s)** |
| --- | --- | --- | --- | --- | --- |
| 1  (*Primary*) | Contraceptive use | Binary indicator that equals 1 if: a) they abstained from both vaginal and anal sex in last 3 months OR b) the number of times they used a condom) during vaginal sex is equal to the number of times they had vaginal sex AND the number of times they used a condom during anal sex is equal to the number of times they had anal sex in the last 3 months. (*Note: r*esponses will be summed across main and casual sex partners)  Or, that equals 0 if: a) the number of times they used a condom during vaginal sex is less than the number of times they had vaginal sex in the last 3 months OR the number of times they used a condom during anal sex is less than the number of times they had anal sex in the last 3 months. (*Note: r*esponses will be summed across main and casual sex partners)  Example items:  In the past 3 months have you had consensual vaginal sex, even once, with a main or casual sex partner? A main sex partner is someone with whom you have an ongoing sexual relationship, with a commitment to each other—like a boyfriend, girlfriend or a steady partner. A casual sex partner is someone with whom you have sex on a casual basis, including as a “hook up” or some people might call it “friends with benefits” [Yes, with a main sex partner; Yes, with casual sex partner; Yes, with main and casual sex partners, No]  During the past 3 months, how many times did you have consensual vaginal sex? Vaginal sex is a penis in a vagina. How many times did you or your partner use a condom during consensual vaginal sex? [Number of Times with Main Sex Partners in the past 3 Months] | Full sample | This outcome measure is a single-item dichotomous measure of behavior | The measure was adapted from Family and Youth Services Bureau (FYSB) Personal Responsibility Education Program (PREP): Promising Youth Programs Core. (Anderson et al., 2021; Office of Management and Budget, n.d.) [50] |
| 1 (Secondary) | Contraceptive use | Same as for primary, but as measured on 9-month post-intervention follow-up survey. | Full sample | See above for primary outcome | See above for primary outcome |
| 2  (Secondary) | Sexual activity and contraceptive use | Number of times condomless vaginal or anal sex in past 3 month (across main and casual sex partners)  See example items for the primary outcome. | Full sample | These outcomes measures are single-item count measures of behavior and will be analyzed individually and will not be combined into one or more scales. Under PREP standards, these measures are considered reliable and do not require documented reliability via a published source. | Anderson et al., 2021 [50] |
| 3  (Secondary) | Self-efficacy | Selected Sub-Scales in Sexual Consent Scale, Revised:   - Positive attitude toward establishing consent (12 questions)   Example items:  Using the following scale, please select the response that best describes how strongly you agree or disagree with each statement. Remember, there are no right or wrong answers, just your opinions. Sexual consent is saying "yes" under your own free will to participate in any type of sexual activity. [Strongly disagree, Disagree, Neutral, Agree, Strongly Agree]  I feel that sexual consent should always be obtained before the start of any sexual activity.   - Sexual consent norms (5 questions)   Example item: I believe that sexual intercourse is the only sexual activity that requires explicit verbal consent. (Reverse coded) | Full sample | Overall internal consistency for the instrument was α = 0.87; internal consistency for the pertinent subscales:   - Positive attitude toward establishing consent (α = 0.84) - Sexual consent norms (α = 0.67) | Humphreys & Brousseau, 2010 [51] |
| 4  (Secondary) | Self-efficacy | Sub-scales in Sexual Communication Self-Efficacy Scale:  Example items:  When communicating about sex with a partner, how easy or difficult would it be for you to…….? [Very difficult, Difficult, Easy, Very Easy]   - Contraception communication (3 questions)   Talk about whether a condom is on correctly?   - Negative sexual messages (4 questions)   Tell them if a certain sexual activity makes you uncomfortable?   - Positive sexual messages (6 questions)   Tell them that a sexual activity feels good?   - Sexual history (3 questions)   Ask if they are having sex with other people?   - Condom negotiations (3 questions)   Demand that a condom be used? | Full sample | Overall internal consistency for the instrument was α = 0.93 with a range of 0.82 to 0.89 for the various subscales | Quinn-Nilas et al., 2016 [52] |
| 5  (Secondary) | Attitudes/  Beliefs | Selected Sub-Scales in Multidimensional Condom Attitudes Scale:  Example items:  How much do you, personally, agree or disagree with each statement below. By contraceptives we mean any type of birth control method that is used for the purpose of preventing pregnancy. [Strongly disagree, Disagree, Neutral, Agree, Strongly Agree]   - Reliability and effectiveness (4 questions)   I think condoms are an excellent means of contraception. | Full sample | Internal consistency for the pertinent subscales:   - Reliability and effectiveness (α = 0.75–0.86) | Helweg-Larsen & Collins, 1994 [53] |
| 6  (Secondary) | Attitudes/  Beliefs | Selected Measures from the Contraceptive Attitude Scale (11 questions; adapted)  Example item:  How much do you, personally, agree or disagree with each statement below. By contraceptives we mean any type of birth control method that is used for the purpose of preventing pregnancy. [Strongly disagree, Disagree, Neutral, Agree, Strongly Agree]  Using birth control/contraceptives is a way of showing that you care about your partner. | Full sample | Test-retest reliability is *r* (166) = 0.88, *p* <.001 | Kyes, 1998 [54] |
| 7  (Secondary) | Equity and power balance in relationship | Relationship Control Subscale (12 questions) and the Decision-making Dominance Subscale (7 questions) from the Sexual Relationship Power Scale-Modified  Example item:  This question provides a list of things that might describe your most recent partner. Please tell us how closely this describes your most recent partner: [Strongly disagree, Disagree, Neutral, Agree, Strongly Agree]  Relationship Control Subscale:  My partner tells me who I can spend time with. (Reverse code)  Decision-making Dominance Subscale:  When my partner and I disagree, they get their way most of the time. (Reverse code) | Full sample | Internal consistency for the Relationship Control Subscale was α = 0.84 | Pulerwitz et al., 2000 [55] |
| 8  (Secondary) | Knowledge | *Count of % correct of included knowledge items:*  Example item:  The next questions ask about your knowledge of your health care rights related to sexual and reproductive health. How much do you agree or disagree with each statement. [Strongly disagree, Disagree, Neutral, Agree, Strongly Agree]  I have a right to get sexual and reproductive health information when I want it.   - Knowledge of health care rights related to sexual and reproductive health (4 questions) - Knowledge of clinic or doctor in the community where teens can get sexual and reproductive health information and services (1 question) - Comfort in going to a clinic to be tested for STIs or HIV (1 question) - Certainty of going or helping a friend go to a clinic for an STI test (1 question) - Knowledge of where to go to get birth control methods (1 question) | Full sample | Items will not be scaled but will be treated as a count of the number correct over the number of items included. | Anderson et al., 2021; [50] Coyle et al., 2021; 56] Office of Management and Budget, n.d. |
| 9  (Secondary) | Contraceptive use | Condom experiences in the previous 3 months (count of 8 experiences)  Example item:  The next questions ask about condoms. Think about all the times you’ve used a condom in the past 3 months for vaginal or anal sex. [No; Yes, one time; Yes, more than one time; Decline to answer]  In the past 3 months, did a condom break during sex? | Full sample | This outcome measure is a single-item count measure of behavior. Under PREP standards, these measures are considered reliable and do not require documented reliability via a published source. | Coyle et al., 2021 [56] |
| 10  (Secondary) | Knowledge | A measure of the % of correct answers comprised from 4 survey questions related to knowledge of educational rights and programs for youth in foster care going to college or trade school (3 questions)  Example item:  The next question asks about the rights and programs available to support youth in foster care going to and succeeding in college and trade school. Mark No, Yes, or Don't Know for each one based on what you think. [No, Yes, Don’t know]  Many California colleges have Guardian Scholar Programs to help current and former foster youth once they arrive on campus. | Full sample | Items will not be scaled but will be treated as a count of the number correct over the number of items included. | N/A |
| 11  (Secondary) | Knowledge | A measure of the % of correct answers comprised from 3 survey questions on the rights and opportunities available to support youth in foster care with managing and earning money (3 questions)  Example item:  The next question asks about the rights and opportunities available to support youth in foster care with managing and earning money. Mark No, Yes, or Don't Know for each one based on what you think. [No, Yes, Don’t know]  Foster youth have a right to open a bank account unless a judge or a specific case plan says otherwise. | Full sample | Items will not be scaled but will be treated as a count of the number correct over the number of items included. | Danes & Haberman, 2007 [57] |
